# Retirement Age and the Age of Onset of Alzheimer’s Disease: Results from the ICTUS Study

**DOI:** 10.1371/journal.pone.0115056

**Published:** 2015-02-25

**Authors:** Catherine Grotz, Luc Letenneur, Eric Bonsang, Hélène Amieva, Céline Meillon, Etienne Quertemont, Eric Salmon, Stéphane Adam, ICTUS/DSA group

**Affiliations:** 1 University of Liège, Psychology of Aging Unit, Liège, Belgium; 2 INSERM, Unit 897, F-33076 Bordeaux, France; University of Bordeaux, F-33076, Bordeaux, France; 3 Maastricht University, Research Center for education and labour force market (ROA), Maastricht, The Netherlands; 4 Centre d'études de Populations, de Pauvreté et de Politiques Socio-Économiques/ International Networks for Studies in Technology, Environment, Alternatives, Development (CEPS/INSTEAD), Esch-sur-Alzette, Luxembourg; 5 University of Liège, Cognitive and Behavioral Neuroscience Center, Liège, Belgium; 6 University Hospital of Liège, Memory Center, Belgium, Liège, Belgium; Hungarian Academy of Sciences, HUNGARY

## Abstract

**Objectives:**

To test whether deferred retirement is associated with delayed onset of Alzheimer’s disease (AD), and, if so, to determine whether retirement age still predicts the age at onset of AD when two potential biases are considered.

**Methods:**

The study sample was gathered from the Impact of Cholinergic Treatment Use/Data Sharing Alzheimer cohort (ICTUS/DSA), a European study of 1,380 AD patients. Information regarding retirement age, onset of symptoms and covariates was collected at baseline whereas age at diagnosis was gathered from the patient’s medical record prior to study entry. Linear mixed models, adjusted for gender, education, occupation, center, country, household income, depression and cardiovascular risk factors were conducted on 815 patients.

**Results:**

(1) The global analyses (n = 815) revealed that later age at retirement was associated with later age at diagnosis (β = 0.31, *p* < 0.0001); (2) once the selection bias was considered (n = 637), results showed that this association was weaker but remained significant (β = 0.15, *p* = 0.004); (3) once the bias of the reverse causality (i.e., the possibility that subjects may have left the workforce due to prior cognitive impairment) was considered (n = 447), the effect was no longer significant (β = 0.06,* p* = 0.18).

**Conclusion:**

The present study supports that there is an association between retirement age and age at onset of AD. However, the strength of this association appears to be overestimated due to the selection bias. Moreover, the causality issue remains unresolved. Further prospective investigations are mandatory in order to correctly address this question.

## Introduction

In the last decades, a growing body of literature has reported a protective association between several proxies of cognitive reserve and the development of dementia among elderly people. In particular, educational attainment[1], active lifestyle (i.e., social, mental, physical engagement)[2–5], bilingualism[6] and complex occupation[7,8] have been repeatedly associated with decreased risk of developing Alzheimer’s disease (AD) and with delayed onset of AD.

The professional setting can be perceived as an “enriched” environment given that, for many individuals, it provides an important source of social support and cognitively stimulating activities, both of which are considered to contribute to cognitive reserve. Leaving the work setting is therefore a major life course transition that causes substantial changes in one’s life and may affect cognitive functioning. This outcome was reported in several studies showing that retirement has an impact on cognition of older adults[9–11]. However, to our knowledge only two recent studies addressed this issue in the context of pathological aging and reported that retirement age is associated with both the risk of developing dementia[12] and the onset of AD[13]. The first ever study in this area was the study conducted by Lupton and colleagues, which revealed that each additional year of employment delayed the onset of AD symptoms by 0.13 years. However, although interesting, this study has major limitations. First, the female population (N = 938) was excluded from the analyses, which considerably reduced the sample size (N = 382) and impeded generalization of the results. A second limitation is that it is still unclear whether the age at onset of AD was based upon a subjective parameter (i.e., reported age at onset of symptoms) or upon a more objective one (i.e., age at diagnosis). Moreover, their study only considered individuals who had already been diagnosed without considering the normal aging population. In addition, they attempted to address the reverse causality issue (i.e., to limit the possibility that people may have retired due to prior cognitive impairment) by excluding individuals, who were diagnosed before retirement. However, due to the particularly long prodromal phase of AD, which lasts more than a decade[14], this strategy is not sufficient to discard the possibility that patients may have been in a prodromal phase of AD that inclined them to retire long before the diagnosis of dementia was made. In other words, the causality issue is not completely resolved. Finally, the selection strategy used by Lupton and colleagues (i.e., considering only AD patients and excluding those who retired after the disease onset) generates a selection bias that leads to an overestimation of the effect of retirement on the onset of AD.

The selection bias observed in Lupton et al.’s study[13] is close to the “healthy worker effect”[15]. The healthy worker effect describes a continuing selection process such that individuals who remain employed tend to be healthier in comparison to those who are retired. In the context of Lupton et al.’s study, the selection bias stems from the fact that as all subjects had already been diagnosed with the disease, and that those who had been diagnosed before retirement were excluded from the analyses, subjects who took later retirement were necessarily those who were diagnosed at a later age. In more concrete terms, an individual who retired at 60 years of age could only be diagnosed after this age, whereas an individual who retired at 80 years of age could only be diagnosed after the age of 80.

In this context, the aim of the present paper was to study the association between retirement age and age at onset of AD (i.e., onset of first symptoms and diagnosis of AD) by taking into consideration the two biases (i.e., the selection bias and the bias of the reverse causality) that may explain the results in favor of the cognitive reserve hypothesis. To that end, we first examined whether deferred retirement is associated with delayed age at onset of AD in the whole sample (i.e., without the inclusion of patients who developed dementia before retirement). Then, we tested whether this association persists when the two biases are taken into account. In order to control for selection bias, we restricted our analyses to subjects who retired at or before 65 years of age. To address the issue of reverse causality, we re-ran our analyses on a subsample of patients who retired at or before 65 years of age and were diagnosed after the age of 75.

## Materials and Methods

### Study design and patient selection

We reported baseline data of 815 patients diagnosed with mild to moderate AD from the two-year longitudinal observational ICTUS/DSA study (Impact of Cholinergic Treatment Use/Data Sharing Alzheimer).

A detailed methodology of the ICTUS study has been previously described by Reynish et al.[16]. Briefly, the ICTUS study aimed to examine the natural history of AD, its treatment patterns and the AD’s socioeconomic impact in Europe. The study was approved by the ethical committee of the Toulouse University Hospitals (coordinating center) and/or local centers and national ethical committees according to the principles embodied in the Declaration of Helsinki.

Patients with probable AD (n = 1,380) were recruited between 2003 and 2005 from 29 specialist outpatient clinics of hospitals in 12 European countries (i.e., France, Switzerland, Italy, Spain, Greece, Germany, Belgium, Romania, UK, The Netherlands, Sweden, and Denmark). The study favored the inclusion of patients who had received recent diagnosis of probable AD (<12 months). This was possible for 95% of cases. All centers were members of the European Alzheimer’s Disease Consortium (EADC Website: www.eadc.alzheimer-europe.org): “a fully functional network of clinical centers with standardized methodologies in the domain of AD management and significant practical experience in carrying out clinical research in this domain”[16]. Therefore, the patients included in the ICTUS study are representative of AD patients followed-up in expert memory clinics across these European countries.

More specifically, the recruitment process was as follows. Patients were referred to the clinical center through different sources (e.g., self-referral, physician). Because of the observational nature of the study, patients’ type of referral did not interfere with their eligibility to participate in the study. In case where a subject met inclusion criteria (e.g., AD probable, initially non-institutionalized), the neurologist invited the patient to participate in the study. All patients and their caregivers received information regarding the study. After a certain period granted to discuss the implications of study enrollment, they provided written informed consent. At inclusion, all subjects received a comprehensive clinical and neuropsychological assessments regardless of whether or not this was their first consulting visit to the memory clinic. The caregiver attended the clinical visit to verify the information provided by the patient.

As regard the present study, it excluded individuals who had reported never having worked, those who had definitely left the labor force before the age of 50, individuals who remained in the workforce and those who retired after the onset of the disease (i.e., patients who reported symptoms and/or were diagnosed). All observations with missing values regarding the outcomes and explanatory variables were discarded from the analysis.

### Outcome variables

Information regarding age at onset of cognitive impairment was gathered from patients and their caregivers (i.e., how old were you at the onset of symptoms?) by a multidisciplinary team (e.g., physicians, neuropsychologists, neurologists) during the first clinic visit.

In order to be eligible, subjects had to be diagnosed with probable AD according to the National Institute of Neurological and Communicative Disorders and Stroke-Alzheimer’s disease and Related Disorders Association criteria (NINCDS-ADRDA criteria) and were required to have a Mini-Mental State Evaluation (MMSE) score between 10 and 26. In the present study, age at diagnosis was recorded by a neurologist/psychiatrist/geriatrician and referred to the date on which the results and conclusion of the different tests administered to the patient showed that all diagnosis criteria were fulfilled.

### Explanatory variables and covariates

Explanatory variables and covariates were recorded by means of a structured questionnaire during the inclusion visit.

An individual was considered as retired if he/she reported not working for pay (information provided by the ICTUS study). Retirement age was considered as a continuous variable in the regression analyses.

Several variables were considered *a priori* based on the literature as potential confounding factors and controlled for in the statistical analyses. Covariates taken into account were gender (with the female population as reference category), educational attainment, occupational status, country (treated as random effect in the regression analyses), clinical center (treated as random effect in the regression analyses), household income, and prior history of diabetes, hypercholesterolemia, hypertension, ischemic heart disease, depression and stroke. Educational level was expressed in number of completed years of formal schooling. Life time occupation was based on a 10-point scale (categorical variable): (1) higher-grade professional (the reference category), (2) lower-grade professional, (3) routine non-manual employee, (4) small proprietor, artisan with employees, (5) small proprietor, artisan without employees, (6) farmer or smallholder, (7) lower-grade technician, (8) skilled manual worker, (9) semi- and unskilled manual worker, (10) agricultural or other worker in primary productions. Household income was categorized as follows: (1) less than 750 euros (the reference category), (2) between 750 and 1500 euros, (3) between 1500 and 2000 euros, (4) between 2000 and 3000 euros, (5) more than 3000 euros, (6) unknown. Self- (and/or caregiver-) reported history of diabetes, hypercholesterolemia, hypertension, ischemic heart disease, depression and stroke were binary variables. This latter information was further supported by the patient’s medical record, which provided the year of diagnosis for each of the cardiovascular and depression variables. Only health conditions preceding the diagnosis of dementia were considered.

### Data analysis

**Step 1**. To assess the relationship between age at retirement and age at onset of AD (i.e., first symptoms and diagnosis), linear mixed regression analyses were performed on the entire sample (n = 815). Univariate analyses were first performed to examine the association between each of the dependent variables (i.e., age at first symptoms/diagnosis) and retirement age. Then, linear mixed regression analyses were conducted controlling for the effects of confounding factors (see above). All subsequent analyses were adjusted for these confounding factors.

**Step 2**. To determine whether this association could be explained by a selection bias, the analyses were carried out on a subsample of subjects who retired between 50 and 65 years old and who were diagnosed after the age of 65 (n = 637). By doing so, we reduced the selection bias given that the subjects who may have created a spurious association between retirement age and age at onset of AD were withdrawn from the study. The choice to restrict the analyses to individuals who retired before the age of 65 stems from the fact that in all European countries, 65 is the highest legal age of retirement.

**Step 3**. Finally, to reduce the reverse causality bias (i.e., to ensure that the subjects were not in the prodromal phase of AD and did not retire due to early symptoms), only subjects who retired before the age of 65 and who developed AD after the age of 75 were considered (n = 447). Thus, the possibility that subjects left their occupation due to early cognitive symptoms seems less probable.

The SAS version 9.2 (SAS Institute, Inc., Cary, North Carolina) was used for all analyses. A *p* value < 0.05 was considered significant.

## Results

### Characteristics of the sample

**Fig 1:**
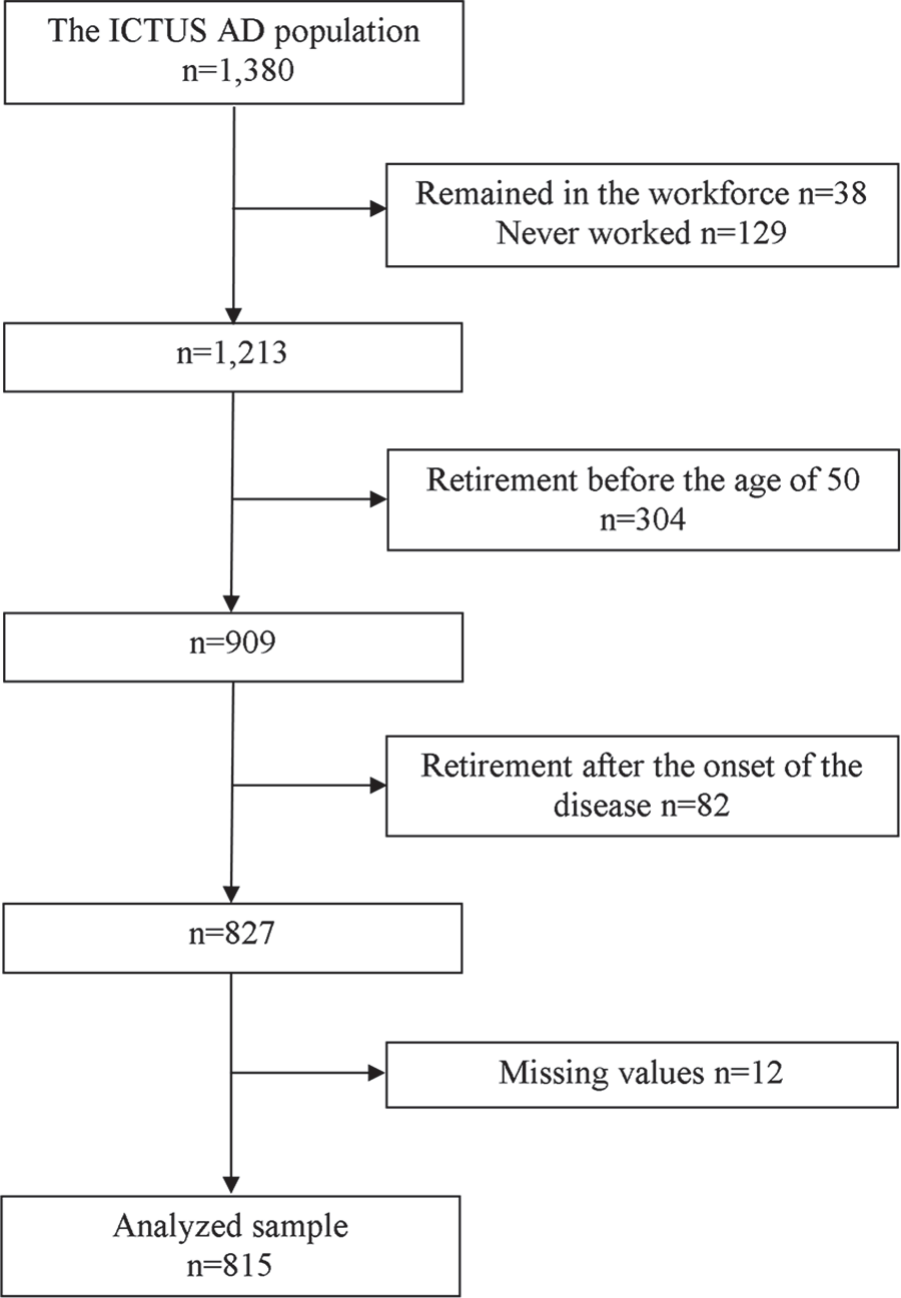
Flowchart of participant selection for the analysis.

Of the 1,380 AD cases, the selection procedure has led to a study sample of 815 patients (see Fig. 1).

The characteristics of the sample (e.g., sociodemographic variables, health status variables) and baseline differences between AD patients discarded from the analyses (n = 565) and those who remained in the study sample (n = 815) are shown in Table 1.

**Table 1:** Study sample characteristics.

	Included sample	Excluded sample	p*
	(N = 815)	(N = 565)	
Age of patients	77.4 (6.2)	74.6 (9.2)	<.0001
Retirement age^a^ Mean (SD)	61.3 (5.1)	45.1 (16.3) (n = 246)	<.0001
Age at onset^b^ Mean (SD)	74.9 (6.2)	71.5 (9.5)	<.0001
Age at diagnosis^c^ Mean (SD)	77.1 (6.2)	74.2 (9.3)	<.0001
MMSE^d^ score at first clinic visit Mean (SD)	20.6 (3.8)	20.1 (4.1)	0.0149
Years of education Mean (SD)	8.1 (4.7)	7.8 (4.5)	0.3824
Sexe-Male n (%)	383 (47.0)	102 (18.2)	<.0001
Diabetes n (%)	102 (12.5)	59 (10.5)	0.2620
Hypercholesterolemia n (%)	186 (22.8)	158 (28.3)	0.0227
Hypertension n (%)	309 (38.0)	221 (39.5)	0.5737
Ischemic Heart disease n (%)	121 (14.9)	62 (11.1)	0.0429
Depression n (%)	198 (24.3)	123 (22.0)	0.3242
Stroke n (%)	61 (7.5)	48 (8.6)	0.4677
Income group of the patient’s household n (%)	-	-	0.1517
Less than 750 euros	226 (27.7)	124 (22.1)	-
Between 750 and 1500 euros	221 (27.1)	154 (27.5)	-
Between 1500 and 2000 euros	105 (12.9)	83 (14.8)	-
Between 2000 and 3000 euros	81 (9.9)	52 (9.3)	-
More than 3000 euros	38 (4.7)	25 (4.5)	-
Unknown	144 (17.7)	122 (21.8)	-

Abbreviation: MMSE = Mini-Mental State Examination.

^a^ Age at which patients stop their remunerated employment.

^b^ Age at which symptoms were first reported by patient or caregiver.

^c^ Age at which the diagnosis of probable AD was made.

^d^ Score out of 30.

* Estimated from chi-square test or Student t test.

The number of years of education, the frequency of diabetes, hypertension, stroke, household income and depression were not significantly different in the two groups. However, excluded subjects were younger, retired at a younger age (25% of the excluded subjects who were professionally active retired before the age of 50), had a slightly lower MMSE score at inclusion and had a higher frequency of hypercholesterolemia but a lower rate of ischemic heart disease. They also had a lower age at diagnosis and a lower age at onset of first symptoms, which is expected since those excluded refer to young cases of AD who were diagnosed before retirement (14.6% of those excluded developed dementia before retirement and 9.8% before turning 60) (see Table 1).

Of the 815 patients, 383 were male (47%). Mean retirement age was 61.3 years (sd = 5.1). Age at diagnosis and age at onset of symptoms followed a Gaussian distribution with a mean age of, respectively, 77.1 (sd = 6.2) and 74.9 (sd = 6.2). Mean MMSE score at the first clinic appointment was 20.6 (sd = 3.8).

### Association between retirement age and age of onset of AD

**Fig 2:**
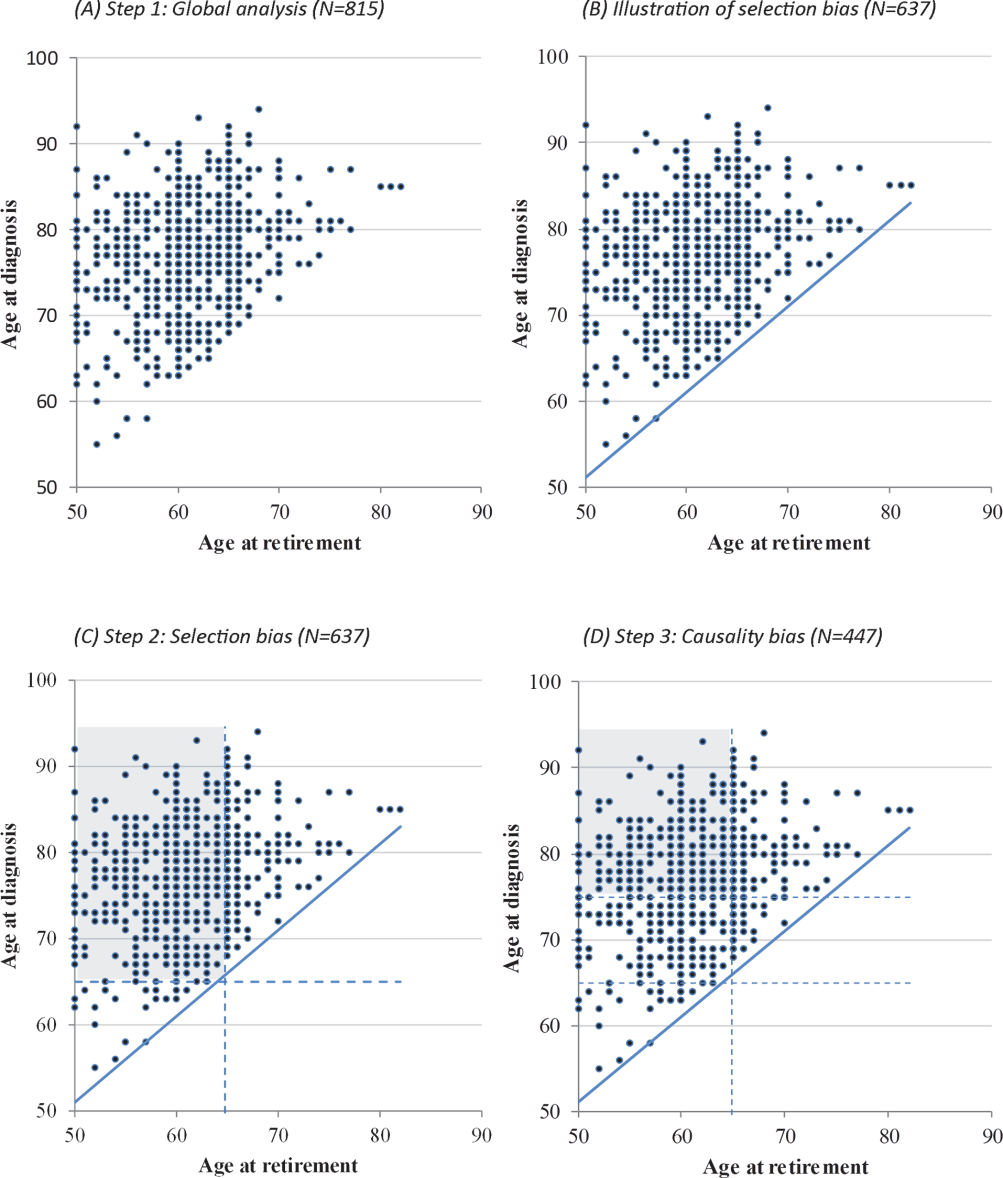
Distribution of the age at diagnosis of Alzheimer’s disease according to age at retirement (N = 815).

**Step 1**. Fig. 2A displays the age at diagnosis as a function of the age at retirement in the sample of 815 subjects. A positive association was observed between the age at retirement and the age at diagnosis (β = 0.33, *p* < 0.0001) and a similar association was found for the age at onset of first symptoms (β = 0.30, *p* < 0.0001). Controlling for potential confounding factors did not change the intensity of the associations: deferred retirement was associated with delayed age at diagnosis (β = 0.31, *p* < 0.0001) and delayed age at onset of symptoms (β = 0.30, *p* < 0.0001). In other words, each additional year of employment delayed the age at onset of symptoms by 0.30 years and the age at diagnosis by 0.31 years. Fig. 2B also shows that due to the selection of subjects who developed dementia after retirement, no data are observed in the lower right-hand side of the chart, suggesting that the subjects who retired later in life influence part of the observed association. As mentioned earlier, a patient who retired at 60 years of age could only develop AD after this age whereas a patient who retired at 80 years of age could not develop AD before turning 80. Therefore, the patient who retired later necessarily has a greater probability of being diagnosed at a later age in comparison to the patient who retired sooner.

**Step 2**. In order to limit this selection bias, we selected a subsample of 637 subjects who retired at or before the age of 65 and who did not develop AD before the age of 65, thus restricting the analysis to the heart of the scatterplot (see shaded area in Fig. 2C). This way, we removed the influence of subjects who retired later (these individuals are also those who developed dementia at a later age). In the multivariate analyses, the age at retirement was still associated with the age at diagnosis, even though the association was weaker (β = 0.15, *p* = 0.004). A similar result was found with the age at first symptoms (β = 0.15, *p* = 0.007).

**Step 3**. A last analysis was performed in order to determine whether the association between retirement age and age at onset of AD could be explained by a reverse causality phenomenon. Among the 447 subjects who retired before the age of 65 and who developed AD at least 10 years after retirement (see shaded area in Fig. 2D), the association between retirement age and age at diagnosis was weaker (β = 0.06) and no longer significant (*p* = 0.18).

## Discussion

The association between retirement age and age at onset of AD (i.e., onset of symptoms and diagnosis) was examined using data collected from baseline information in a sample of 815 patients with probable AD from the longitudinal observational European ICTUS study. Our first aim was to carry out the analyses using a similar strategy to that of Lupton and colleagues[13] (i.e., the first study that addressed this issue in pathological aging). Consequently, our analyses were conducted on AD patients who were diagnosed after retirement. The empirical results highlight that each additional year of employment delayed the age at onset of symptoms by 0.30 years and the age at diagnosis by 0.31 years. Such results were obtained after controlling for gender, education, occupation, country, clinical center, income household, and prior history of diabetes, hypercholesterolemia, hypertension, ischemic heart disease, depression and stroke. These results are consistent with those of Lupton and colleagues[13], as they show that each additional year of employment delayed the onset of dementia symptoms by 0.13 years. In Lupton et al.’s study, the exclusion of patients who were diagnosed before retirement led the authors to assume that it is retirement that has an impact on the onset of AD and not the other way around. Nonetheless, this modality of exclusion of patients is not sufficient to exclude the possibility that patients may have had cognitive impairment that caused them to retire (i.e., reverse causality).

Furthermore, this strategy of patient selection (i.e., AD patients who were diagnosed after retirement) creates a selection bias that leads to an overestimation of the effect of retirement on the onset of AD. In Lupton et al.’s study[13], this selection bias stems from the fact that due to their selection of patients, those who retired later were necessarily those who developed AD at a later age.

Within this context, the second aim of this paper was to replicate similar analyses, taking into account these two biases (i.e., the selection bias and the bias of the reverse causality).

To this end, we first took into consideration the selection bias by restricting our analyses to subjects who retired before the age of 65 and did not develop AD before 65 years old. By doing this, we were still able to find a significant association between retirement age and age at onset of AD, but to a lesser degree. More specifically, we found that each additional year of employment delayed the age at onset of diagnosis and the age at first symptoms by 0.15 years. Therefore, taking into account the selection bias reinforces the hypothesis of an association between deferred retirement and further age at onset of AD, as observed in Lupton et al.’s study[13]. However, even though this association persists and remains significant, it is still plausible that patients left the workforce due to prior cognitive impairment, with several studies showing that the prodromal phase of AD lasts more than a decade[14,17,18]. Hence, the same analysis was conducted after exclusion of individuals who were diagnosed within 10 years after retirement. This latter analysis shows that the association between retirement age and age at onset of AD is reduced by half and is no longer significant. Although a lack of power could be argued due to the considerable reduction of our sample size, it still comprises 447 subjects. This finding highlights that the conclusion of Lupton and colleagues[13] is probably overstated. Indeed, the selection of AD patients who developed the disease before retirement (in order to ensure that people did not leave the workforce due to prior cognitive impairment) only leads to an automatic association between a later age at retirement and delayed onset of AD. As a result, it was critical to use similar design in order to show that this selection strategy induces a selection bias, which leads to an overestimation of the effect of retirement. Thus, our study clearly points out that caution is needed when studying the effect of retirement on AD patients’ health outcomes. Based on these observational data, it is still unclear whether it is retirement that has an impact on the onset of AD or whether it is cognitive impairment that inclines individuals to retire.

Nevertheless, several studies in “healthy” aging suggested that it is retirement as such that has an impact on the cognitive functioning of elders[9,10,19]. First, a country level analysis highlighted that cognitive score of elders was better in countries in which eligibility age for retirement benefits tended to be higher (e.g., 65 years of age in Sweden) compared to those where it tended to be lower (e.g., 60 years of age in France). Given that countries do not set the eligibility age for retirement based on the cognitive vitality of individuals, the authors argue that this finding is a strong argument in favor of the role of retirement on cognition. Second, longitudinal data from the Health and Retirement Study (HRS) shows that the curves of the rate of retirement and memory decline can be overlaid, with a lag of about one year. For instance, while the rate of retirement in the United States is higher at 62, 65, and 66 years of age, a significant cognitive decline with a lag of about one year at the ages of 63, 66, and 67 is observed. These data suggest that retirement is not a consequence of prior cognitive decline, but rather that departure from the workforce causes a decrease in cognitive performance with a delay of about one year post-retirement.

Although these studies highlight that retirement plays a role in cognitive functioning of older adults, it is indisputable that in the context of pathological aging, there is a lack of empirical evidence. Further prospective studies (including both subjects with dementia and “healthy” older adults) are thus mandatory in order to better address the effect of retirement on the onset of AD. Before concluding that deferred retirement has a protective effect on the aging population’s health, some issues have to be addressed. For instance, future studies should seek to determine whether the association between retirement and cognition is direct and/or whether some intermediate variables such as social networks or depression influence this association. Indeed, work is known to increase social interaction and sense of self-efficacy[20], both variables associated with cognitive functioning of elders[21,22] and decreased risk of dementia[5,23]. Above all, further studies should test whether the impact of retirement on cognitive functioning depends on different types of retirement (e.g., voluntary versus involuntary departure), on participation in cognitively stimulating activities, and/or on occupational activity undertaken while employed (e.g., physical versus intellectual work). For instance, some studies have reported that cognitively stimulating occupation during adulthood and active lifestyle are associated with lower age-related cognitive decline[24,25] and also with lower risk of developing dementia[4,7,26]. Nonetheless, a longitudinal study found differential cognitive trajectories prior and following retirement in individuals with high complexity of work. Specifically, high complexity of work with people was associated with better verbal performances during employment followed by a faster rate of cognitive decline after retirement on spatial skills[27]. Therefore, further studies should seek to better understand the relationships between complexity level of occupation during adulthood and retirement and its influence on subsequent trajectories of cognitive decline.

In conclusion, our results support that, in the context of pathological aging, the association between retirement age and age at onset of AD requires further studies, with prospective design, to better assess the impact of retirement on AD patients’ health.
